# Using community‐based participatory research to contextualize Latino exposure to community violence: A mixed qualitative and spatial analysis approach

**DOI:** 10.1002/ajcp.70003

**Published:** 2025-07-24

**Authors:** Kyle C. Deane, Maureen T. S. Burns, Maryse H. Richards, Catherine DeCarlo Santiago, Ogechi “Cynthia” Onyeka, Amanda White, Felix K. So

**Affiliations:** ^1^ Shriners Children's Chicago Chicago Illinois USA; ^2^ Rosalind Franklin University of Medicine and Science North Chicago Illinois USA; ^3^ Loyola University Chicago Chicago Illinois USA; ^4^ Brave Minds Psychology Center for Child Anxiety Murrieta California USA; ^5^ University of California Los Angeles California USA

**Keywords:** community based participatory research, community violence, GIS, Latino youth, resilience, school setting

## Abstract

While the relationship between community violence exposure and maladaptive outcomes has been established, the dynamic between violence exposure and resilience factors in youth is not well understood. The current study utilizes a community‐based participatory research (CBPR) framework and employs a novel mixed‐methods approach integrating quantitative geographic information systems (GIS) data and semi‐structured qualitative focus groups to examine violence exposure, family functioning, and neighborhood characteristics, such as community assets, as experienced and reported by Latino adolescents. Participants (*N* = 40; age 12–18) included Mexican American youth residing in an urban area and were recruited based on their involvement in a youth organization. The youth‐made maps and focus groups revealed that participants identified friends and family, social capital, and community engagement as safe and protective. However, the characterization of schools was more complicated and inconsistent. While schools appear to be sources of refuge and places to process neighborhood stressors for some youth, exposure to violence within and around school made them unsafe for others. Future studies and interventions, especially school safe passage programs, should consider a similar CBPR mixed‐methods approach due to the precision of the GIS data and the youth voice brought by the qualitative methods.

## Key highlights

- Integrates GIS data and youth‐made maps for precise insights on violence exposure and safety.
- Utilizes CBPR to amplify Latino youth voices on family, community, and neighborhood resilience.
- Finds schools as both an area of risk and refuge, highlighting nuanced youth experiences.

Over the past two decades, researchers have continued to clarify the complicated mechanisms by which neighborhood characteristics influence children and adolescents' development and psychosocial functioning (for a review, see Lund et al., 2018). Youth living in highly stressful environments, impacted by systemic oppression, poverty, violence, poor nutrition, unemployment, and limited resources, are at significant risk for deleterious physical and mental health outcomes (Shern et al., 2016), constituting a demanding public health concern, that disproportionately effects youth of color, including Latino youth. While there is ample research demonstrating the interrelated nature of violence, maladaptive outcomes, and other risk factors, these relationships are less understood in the context of various resilience factors, including social support networks, family functioning, and community assets (Kaynak et al., 2011; Quinlan‐Davidson et al., 2021). The current study will attempt to explain these interrelations through the utilization of a mixed‐methods, place‐based paradigm emphasizing a youth perspective among adolescents living in a low‐income, high violence neighborhood in Chicago, IL.

## EXPOSURE TO COMMUNITY VIOLENCE

One neighborhood characteristic that has received considerable attention in the literature is exposure to community violence. Community violence can be broadly conceptualized as acts of interpersonal behavior that threaten, attempt, or accomplish the intentional infliction of psychological or physical harm committed by individuals not intimately related to the victim (The National Child Traumatic Stress Network, 2016). While rates of violent crime have declined in the United States over the past decade, children's rate of violence exposure as a witness or victim remains alarmingly elevated within many urban environments (Reed et al., 2014). Moreover, crime data indicate that youth of color living in urban, economically disadvantaged communities are disproportionately exposed to this form of violence (Deane et al., 2016), with an estimated half of all youth in these environments experiencing exposure (Finkelhor et al., 2015).

In addition to adversely affecting mental health functioning, violence in these communities can directly shape how youth perceive and interact with their neighborhood. Lee et al. (2017) reported that perceived crime was the greatest predictor of neighborhood satisfaction among residents. Community violence also reduces walkability in neighborhoods, resulting in a barrier to many students' commute to school (Wiebe et al., 2013). Across the United States, 5.5% of high school students reported not attending school one or more days in a month given a perceived lack of safety at school or on their way to or from school (Eaton et al., 2008). There have been few investigative efforts dedicated to understanding the danger from community violence that adolescents may experience as they traverse between their home and school environments.

Within neighborhoods with high levels of violence, several aspects remain unclear, including the location of violence exposure as well as which places and situations represent a refuge. Indeed, the locations that youth may seek for protection may also be the same areas in which violent incidents occur. For example, among a sample of urban youth engaging in a time‐sampling study, a large portion of adolescents reported witnessing a violent act taking place on or near school grounds (Richards et al., 2015). There is a particularly wide gap in research examining these variables of violence exposure among Latino youth (Reingle et al., 2013). While violence in Latino communities is not solely attributable to gang activity (Santacrose et al., 2021), the presence of gangs in neighborhoods appears to place youth at greater risk for exposure given risk of initiation as well as closer proximity to gang activity (Howell, 2011).

On the whole, measuring a multidimensional element like community violence remains a challenge. There is no consensus on the weight of factors including the observer, their proximity to violence, or the magnitude of violent events (Kennedy & Ceballo, 2014). Furthermore, the relationship between youth's exposure to community violence and their reported perception of violence in their neighborhood is complicated. While some youth who report experiencing high levels of community violence also perceive their neighborhood as unsafe, some youth who report low levels of violence exposure also perceive their neighborhood to be unsafe (Cammack et al., 2011). It is crucially important to examine the complex relationship between community violence exposure and youth perceptions of neighborhood safety to better examine how resiliency factors, such as social support networks, play a role in buffering negative effects of community violence.

## THE ROLE OF COMMUNITY ASSETS AND SOCIAL SUPPORT NETWORKS

While the negative effects of violence exposure and living in an economically disadvantaged community are clear, not all youth residing in hazardous environments demonstrate equal levels of distress. Indeed, youth show various levels of exposure and adaptation or adjustment, potentially due to a collection of individual characteristics, social support networks, and community assets (Kaynak et al., 2011; Quinlan‐Davidson et al., 2021). Murayama et al. (2012) described social capital, a collection of social resources, as a critical asset for fostering individual and neighborhood well‐being. While physical neighborhood resources, such as schools, parks, libraries, churches, and youth organizations have been linked with neighborhood satisfaction and promotion of positive adjustment among adolescents (Hart & Mueller, 2013), informal social support networks, such as family, friends, and recreational areas also likely play a role in the promotion of psychosocial well‐being. Indeed, the amount of time spent with family and friends appears to protect against violence exposure among youth (Goldner et al., 2011).

One important element of a youth's social capital is their family, including family support, cohesion, parenting practices, and overall functioning. Raising children in dangerous surroundings marked by crime and violence presents a considerable challenge for parents, and several studies have demonstrated a vast set of strategies that parents utilize to minimize the negative effects of violence exposure or other dangers on their child's development (Leventhal & Dupéré, 2019). These practices include enforcing curfews, chaperoning or forbidding children and adolescents from engaging in certain extracurricular activities (Outley & Floyd, 2002), and spatial restriction (Furstenberg, 1999). In addition to caregiver protective factors, other family functioning attributes have been associated with improved outcomes in the context of toxic stress, including family cohesion (Joos et al., 2019) and perceived family support (Li et al., 2007; Shern et al., 2016). While Latino youth reported increased levels of self‐efficacy in the context of high family cohesion and positive parenting (Leidy et al., 2010), family practices are not homogenous across Latino subgroups or generational immigration status and parenting practices are shown to influence the relationship between exposure to and perception of community violence (Antunes & Ahlin, 2021).

## GEOGRAPHIC INFORMATION SYSTEMS AND MIXED‐METHODS RESEARCH

Though many studies have examined the individual and proximal variables that contribute to psychological and behavioral outcomes, more study is needed of contextual factors, such as neighborhood characteristics, and the mechanisms by which they affect safety. Geographic information systems (GIS) technology has been increasingly used to investigate and analyze neighborhood characteristics in a graphical and accessible manner. There have been few applications of the technology to examine community violence in neighborhoods (Wiebe et al., 2013). GIS has been utilized, however, as an approach in a mixed‐methods paradigm (Keddem et al., 2015), with several studies exhibiting the usefulness of GIS combined with qualitative methods in a mixed‐methods approach (Dennis et al., 2009). GIS offers researchers an opportunity to integrate survey, government, or other authoritative data into a study, even collecting youth‐generated spatial data, which has been rarely employed. The use of a mixed‐methods qualitative and GIS approach may provide further contextual information regarding the links between neighborhood characteristics, violence exposure, and family and peer support.

## COMMUNITY‐BASED PARTICIPATORY RESEARCH

The current study employs a community‐based participatory research (CBPR) approach. CBPR is an approach to health and environmental research that is designed to increase the value of studies for both researchers and the community under investigation (Viswanathan et al., 2004). This type of research involves collaboration between researchers, organization representatives, and community members. As youth are considered “experts in their own lives” (Langhout & Thomas, 2010), and youth reports of perceptions of neighborhood are more reliable than parents' in predicting child outcomes (Byrnes et al., 2007), the use of CBPR with adolescents is well suited to collect accurate youth perceptions. CBPR affords the use of various methodologies (e.g., survey methods, focus groups) to develop a more comprehensive socio‐demographic profile (Daley et al., 2010). Applying this method to the study of violence exposure and neighborhood characteristics is of interest to researchers given increasing awareness of the interrelated nature of a child and adolescent's individual, familial, social, and community contextual levels. In a unique application, we have integrated the CBPR with the GIS by asking the youth to complete maps of their communities.

## CURRENT STUDY

While the aforementioned studies examining violence exposure, social capital, and family functioning provide insight into these variables, they do not incorporate community and youth perspectives that may help to explain the interrelations of these elements within a spatial or qualitative context. Relatively few studies examine variables through a youth perspective, fewer utilize GIS technology to examine youth geographic experiences, and even fewer use a mixed‐methods approach to describe the dialectical relationship between youth exposure to violence and experience of neighborhood. The current study provides a methodological and empirical contribution to the literature on exposure to community violence among Latino youth living in high violence, low‐income neighborhoods. Through the utilization of a mixed‐methods CBPR design, the current study qualitatively and quantitatively examines violence exposure, family functioning and various neighborhood characteristics, including perceptions of neighborhood safety and various protective community assets and social support networks identified by Mexican American youth living in South Lawndale, or “Little Village,” the largest Latino neighborhood in Chicago, IL. The variables of interest were measured and presented by utilizing a mixed‐methods approach consisting of GIS technology along with semi‐structured qualitative focus groups.

The present study seeks to examine three aims and related hypotheses. The first aim is to examine the incidents of violence reported by youth and by the Chicago Police Department (CPD). It is expected that the youth‐made maps and the maps made from CPD crime data will indicate differing or distinct areas of concentrated violence reports, indicating that the youth and police have different perceptions of violence in the community (*hypothesis 1*). The second aim of the study is to examine youth‐reported routes to and from school in Little Village in relation to perceived unsafe areas. It is expected that focus group reports and youth generated maps will indicate that a substantial number of youth traverse a significant distance of perceived unsafe regions and gang territories on their route to school (*hypothesis 2*). Finally, the third aim is to examine and display what youth identify as safe areas and community and social assets in relation to incidents of violence. Youth of all risk and age groups are expected to identify family, friends, libraries, parks, churches, and/or community organizations as important assets in promoting safety, both through the quantitative and qualitative data collection (*hypothesis 3*).

## METHODS

### Participants

A sample of 40 Mexican American youth residing in an urban area aged 12 to 18 (*M* = 16, 50% female) was recruited for a study examining neighborhood perceptions and exposure to community violence. In accordance with CBPR collaborative methodology, outreach workers and school‐based mentors from a Chicago‐based non‐profit violence prevention and community organization, along with academic researchers, jointly developed study aims and identified youth involved in the Chicago‐based organization programming to participate. 56% of youth resided with both parents, 33% lived with either parent, and 11% lived with extended family members. Most parents did not complete high school (77% of fathers, 61% of mothers). Most participant mothers were identified by youth as homemakers (67%) and most fathers worked full time (72%). 88% of youth were U.S.‐born while the remainder were first‐generation immigrants from Mexico.

All youth resided in the Little Village neighborhood, an urban neighborhood on the West Side of Chicago, IL. This neighborhood is characterized by a predominance of Mexican‐American residents, with 75% of the residents identifying as Mexican American (Ready & Brown‐Gort, 2005). Little Village is also comprised largely of low‐income families, with 31% of its residents living below the poverty line (City of Chicago Census Data, 2008‐2012). According to 2014 crime statistics compiled by CPD and the Cook County Medical Examiner's Office, Little Village had the fifth highest number of youth homicides out of 77 community areas in Chicago (RedEye Chicago, 2014). It is an area marked by elevated violent crime rates and gang violence (National Gang Center, 2021). In 2013, the CPD recorded 2,750 crimes in Little Village, over 1,100 of which were violent (City of Chicago, 2016).

To maximize openness to discussion and promote group cohesion, the non‐profit organization staff divided participating youth into five distinct cohorts based on their respective involvement with organization programming. These five separate cohorts comprised youth involved in community mentoring (two separate groups out of the five), academic mentoring and college preparation (another two groups out of the five), and a work experience program. The groups were categorized in terms of overall risk and functioning following completion of data collection based on youth placement in these various groups, academic transcript information, knowledge of social functioning and family environment, and additional demographic information (Deane et al., 2020). Youth in community mentoring programs consisted of two groups labeled as “higher‐risk,” with one group containing the youngest members and the other with known gang members. Youth participating in academic mentoring were separated into two groups classified as “lower‐risk,” with one group in a college preparation program and one group containing youth in less risky home and peer environments. Finally, youth involved in the work experience programming formed the “mixed‐risk” group, containing a larger group of mixed ages and moderate risk level.

### Procedure

Participation in the study was voluntary and youth responses were confidential. Parent or guardian consent and youth assent or consent was received before data collection for each participant. The youth were given a $40 gift following the fourth and final focus group session as an incentive for participation. The youth were enrolled in four separate focus groups of approximately ten youth per group divided based on age groups. Trained research staff conducted four focus group sessions over the course of three to 5 weeks. The scripts were developed to prompt discussion pertaining to youths' perceptions of: (1) neighborhood experiences and characteristics, (2) psychosocial functioning, mental health, and youth empowerment, (3) school and community connectedness, (4) and family and cultural experiences. A trained research team member and staff member of the non‐profit organization conducted each focus group. All sessions were audio recorded and transcribed by members of the research team.

After the focus group meetings, the youth participated in an interactive GIS mapping exercise, using ArcGIS (Esri, 2016) custom online map templates, which involved youth individually identifying various spatial points and areas of interest within the Little Village neighborhood. The youth were asked to produce their own community map of Little Village using a prepared online mapping template, complete with reference locations and a street map overlay. Information presented by the youth from the focus group also informed the collection of spatial data. Informed, in part, by these themes, the following parameters were obtained for each participant: youth's home, friends' homes, hangout locations, youth community programs, gang territories, perceived safe and unsafe territories, their route and mode of transportation to school, and the specific locations of witnessed or experienced violent incidents or other crimes. Each participant created points, lines, and polygons depicting these spatial locations.

### Analytic procedure

#### Spatial data analysis

ArcGIS for Desktop was used to analyze the mapping information gathered from participants. Crime data for the calendar year of 2013 was obtained from the City of Chicago data portal (https://data.cityofchicago.org/) as reported by the CPD. These data were filtered to exclusively include crime that took place in public places using the location parameters of *abandoned building*, *alley*, *bar or tavern*, *church/synagogue/place of worship*, *CTA bus stop*, *CTA train*, *CTA platform*, *commercial/business office*, *driveway*, *hotel/motel*, *library*, *parking lot*, *park property*, *residence porch/hallway*, *residential yard*, *restaurant*, *school*, *sidewalk*, *street*, *vacant lot*, and *vehicle*, while excluding *apartment*, *residence*, and *residence‐garage*. Data were further filtered to include only violent crime exposures, including *armed robbery*, *assault*, *battery*, *child sex abuse*, *criminal damage*, *criminal sexual assault*, *homicide*, and *unlawful use/possession of handgun*. There were 1,209 total violent crime incidents reported by the CPD in 2013 in Little Village. Additional publicly available data, including location of libraries, places of worship, and schools were obtained.

The data from CPD and the youth focus groups were uploaded to ArcGIS over a map of Little Village. A kernel density function, a technique that transforms a set of discrete data points into a continuous surface and gives weight to points based on density, was run on both datasets. This function has been used to objectively compare geographic datapoints in urban settings (Kloog et al., 2009). Then, each data set was divided by its own maximum value, to make the datasets of differing sizes comparable on a scale of 0–1. To examine the difference between the distribution of the two datasets, a raster subtraction function was used. To examine incident proximity correlations to features of interest (i.e., schools and parks), chi‐squared tests for spatial correlations were utilized, in which the expected distribution of violent incidents was created by assuming uniform distribution.

#### Focus group data analysis

Following grounded theory methodology described by LaRossa (2005), the research team used an open, axial, and selective coding procedure. The research team and a senior organization staff member performed preliminary coding of transcripts based on the key areas of interest from the topics discussed by youth in the focus groups. Research team members developed and reviewed the initial coding to determine the breadth of each domain and then expand, condense, or remove initial codes to define a final coding scheme based on team consensus. A trained expert coder from the research team reviewed codes from 20% of all transcripts completed by each coder to ensure inter‐rater reliability of 0.80 and above. Atlas. ti 7.1, the qualitative data analysis and research software, was used to perform content analyses to investigate and analyze themes and information gathered from the semi‐structured focus groups.

## RESULTS

### Spatial analyses

Using GIS ArcMap, youth spatially identified a total of 89 witnessed or experienced violent incidents, 38 youth programs, and 93 areas of social interaction and support within their neighborhood within the past year. Youth mapping input revealed that youth identified friends and family as safe areas in addition to traditional protective community assets (e.g., churches, youth organizations). Indeed, an examination of youth‐inputted text revealed that 44% of safe areas identified were related to these more informal social support networks.

**Figure 1:**
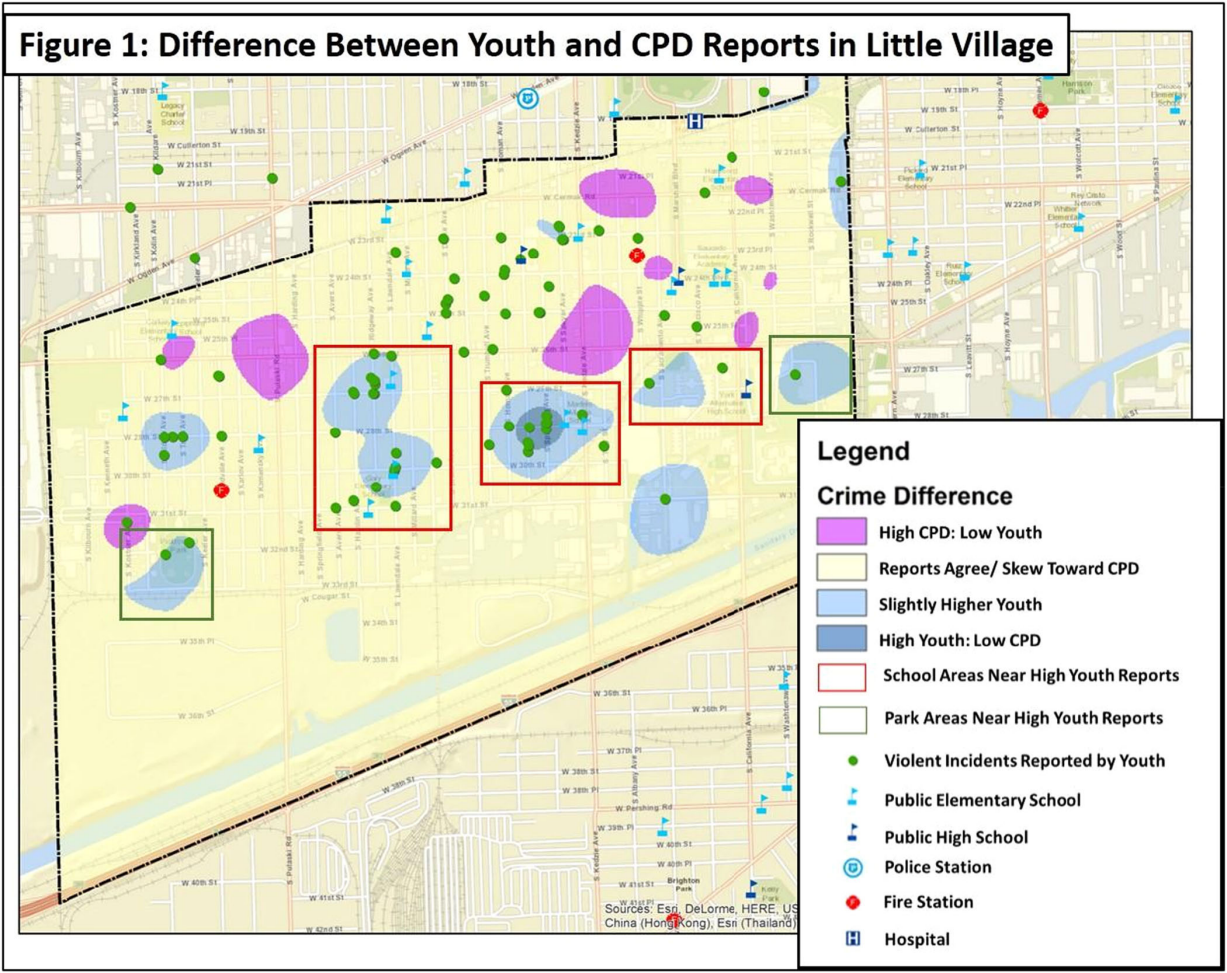
Difference between youth and CPD reported in Little Village.

**Figure 2:**
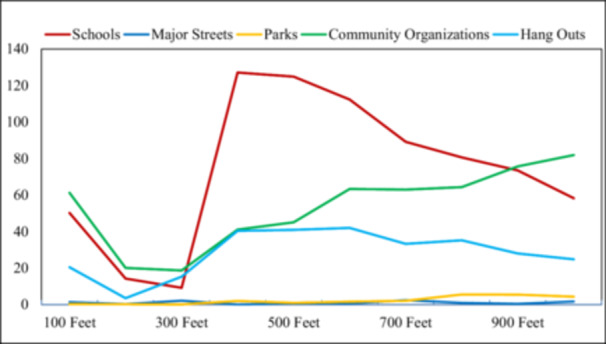
Chi‐squared test of youth‐reported violent incidents by community landmarks.

The first aim of the current study was to compare incidents of violence reported by youth with those reported by CPD. Youth report and CPD report of violence were found to be strongly positively correlated *r*(88) = 0.85, *p* < .00. A “Difference Map” (Figure 1) was created to compare the youth report of violent incidents to the CPD data set. It was made by dividing each data set by its respective maximum value to make the sets comparable on a 0 to 1 scale. Youth reported a higher relative density of violent incidents (indicated by the blue spaces) around schools (highlighted by the red rectangles) and parks (highlighted by the green rectangles) when compared to the CPD report. In contrast, CPD reported a higher concentration of data points near major intersections and commercial areas (as indicated by the purple spaces) when compared to the youth reports. Furthermore, a Chi squared analysis

$$X^{2}=\sum \frac{\left(\mathrm{Observed value}-\mathrm{Expected value}\right)2}{\mathrm{Expected value}}$$

compared an assumed even spread of violent incidents to the youth reports. It found a reported safety buffer zone of 200–300 feet (1–2 city blocks) outside of schools and organized programs, followed by a sharp increase in violence outside these buffer zones, as seen in Figure 2. No significant difference was found around major streets or parks. When compared to the even spread of violent incidents, the CPD reports indicated significantly more points closer to major street and thoroughfares and a less robust spike in violence surrounding schools, in a 250–500 feet zone.

The second aim of the current study was to examine youth‐reported routes to and from school in relation to exposure to violence and perceived safety. Regarding the primary method of transportation for the participants: 67% walk, 3% bike, 9% take the bus, 9% take the train, and 12% ride in a car. To determine youth travel through perceived unsafe or gang territories, the Intersection tool in ArcMap was utilized (Esri, 2016) to generate a geometric intersection of individual youth route and corresponding unsafe or gang regions. Youth generated maps revealed that 53.3% of youth reported traversing through areas that they perceived as unsafe or gang territories on their way to school. Of the youth whose routes intersected with these self‐identified regions, the average proportion of distance within a gang territory was 38.85%, which corresponds to an average of 1,101 feet, and 23.51% proportion of distance on average within perceived unsafe territories, which corresponds to an average of 677.5 feet.

The qualitative analyses demonstrated key themes across three broad areas related to the study hypotheses on resources and exposure to community violence: Community Assets, Social Support Networks and the Routes to and from School. The final aim was to qualitatively examine what youth identify as safe areas and community and social assets in relation to incidents of violence. See Table 1 for illustrative quotations.

**Table 1:** Focus group codes, themes, and selected quotes.

Codes	Themes	Quotes
Community assets	Churches	“I volunteer at the church, Amor de Dios. It's peaceful around there. Gangbangers come there to play basketball, but they never really get out of hand or anything, they just have fun there, so it's peaceful.”
	Schools	“[Fights occur] during all three. Before, after, inside the school.” “She personally pulled me out of class and [asked,] ‘What's going on?’ She knows my name she was super supportive.”
	Volunteer organizations and clubs	“I tried to stay very involved. I'm part of the Dreamer's Alliance – we go out to the community a lot, we try to keep them informed about opportunities that undocumented students and people have.”
	Parks	“I think a lot of people get together [at the park]… A lot of people go there, but there's so many gang members there.”
Social support networks	Home and block	“I think the safest place is your house.” “The people. You know everyone on your block, everyone knows you. So, they don't really mess with you.”
	Friends	“Almost all my friends, they all really mean something good to me. And I'd be ready to give my life for them. And it's the same way for them.”
	Role of family functioning	“I think that's the source of all the problems and all the violence, and all the negative stuff starts with families. I think that the base of everything, the base of neighborhood, the base of city starts with family.” “I think that another way I look at it, is that half of the people that I believe are gang affiliated is because family has forgotten about them and not paid attention to what they really needed.”
Route to school	Avoiding certain areas within the neighborhood	“[My mom] doesn't even want us after school to be on Cermak… Our parents always tell us, ‘I don't want you over there, you guys have no business over there.’ I have friends that live over there, but I can't go over there to hang out.”
	Traveling in groups and group affiliation	“I would tell them it's safe if you're with someone who lives around here, so they could let you know where everything's at.” “Pretty much Little Village is good only if you know people.”
	Safe route program	“I was part of a group called “Mikva.” The whole point of it was to talk about how our school worked and we talked about things that don't work. We also talked about safe and unsafe places in the community also, and we actually decided where to put community watchers in the community.”

### Community assets

#### Volunteer Organizations/Clubs

After‐school activities, such as volunteer organizations and clubs, emerged as common sources of positive involvement within the community for youth, despite community violence, particularly among youth in the lower‐risk, college‐bound group. It was also expressed that these activities may serve to provide positive examples for other youth in the community. One example from the lower‐risk college‐bound group, one participant described the “Mikva” club as addressing their community's safe and unsafe areas to decide on the placement of “community watchers in the community.” Across all groups, other types of clubs and organizations reported by the youth included: El Vejo, Enlace, Caps, and Project Vida, many of which have missions related to reducing violence exposure and its deleterious effects.

#### Churches and places of worship

The influence of church affiliation as a protective factor in relation to community violence exposure was noted among youth in the low and mixed‐risk groups. Youth identified their church and participation in church activities as a peaceful and positive alternative to the socially toxic environments produced by community violence. Participants reported their church as a place where even those affiliated in gang activity can play basketball there and nothing “really gets out of hand,” where students can study safely, and where individuals are available to talk to them and “help them [with] whatever they need.”

#### Schools

Youth across focus groups expressed ambivalent views regarding the safety of schools. Some focus groups highlighted specific concerns regarding safety within and around schools. One youth from the mixed‐risk group described feeling safe in the morning but not while leaving school: “If you're in after‐school and you are coming out late, there are probably gangs around.” Another youth from the higher‐risk, gang‐involved group noted that fights occur during all times of the day, before, during, and after school. Two youth from this higher‐risk group highlighted racial tensions within the school remarking, “The Hispanics, the African Americans, we fight just because they want to go down.” In contrast, while no participant specifically identified schools as safe areas, some groups described teachers and mentors within the school as encouraging involvement in the community, avoidance of gang membership, and providing support following violence exposure. A youth reported that after‐school programs, such as tutoring and community engagement, provided structure and prevented violence.

#### Other community assets and areas

Other regions of the neighborhood were cited by youth as being both valuable assets and areas of potential danger within their community, including parks, hospitals, libraries, and shopping malls. Youth from all risk groups cited various establishments in commercial districts, such as ice cream shops, restaurants, and clothing stores, as being places of congregation, safety, and support. Various responses from the focus groups suggest that public parks were perceived as peaceful areas within the community, while others viewed parks as dangerous areas to avoid. For instance, one participant described neighborhood parks as areas that serve as a distraction from various stressors he experiences. In contrast, another youth described the need to avoid certain parks at night due to known gang associations.

### Social support networks

#### Home/Block

In all groups, regardless of age or risk, youth described the level of perceived safety and security within the context of their home or block. Various focus group transcripts revealed that the characteristics of one's home or neighborhood block were viewed as a significant protective network in relation to community violence. Individuals from the lower‐risk and lower‐risk college‐bound youth described their home as “the safest place.” A topic frequently discussed was protective relations with people on the block. One youth reported on the importance of associating with the right people, noting the “good people” and “bad people” and that “if you follow the right road, you will make it in Little Village.” Alternatively, some youth reported their neighborhood context as unsafe, referring to the presence of gang activity outside of their homes.

#### Friends

Youth described strong protective bonds formed with friends in the community across all risk groups. In response to the question of whether there are friends around the neighborhood who can be considered as family, one participant each from the higher‐risk gang involved group and the lower‐risk college bound group, stated in an almost identical fashion, “I consider [one friend] like family, like we've been knowing each other for a very long time, and… she's like a sister to me. She knows my family, we know each other's families, we just understand each other, trust each other, like a sister.”

#### Role of family functioning

Youth endorsed themes related to family functioning, perceived family support, and family presence with regard to community violence exposure. Within such contexts, youth recognized the role family members (particularly parental figures) serve in either working to protect the youth from violence or the lack thereof due to their absence. Youth from the lower‐risk college‐bound cited family members, such as uncles, aunts, siblings, and parents, as sources of support during times of crisis and as important role models.

### Route to school: Managing risk of violence

Regardless of risk level, concerns regarding traversing the neighborhood and safe passage to and from school were endorsed by all groups. Youth from all groups recounted avoiding certain areas due to fear of violence. One participant described navigating interactions with gang members during her route home, saying, “When I walk out of school… there's always a bunch of gangbangers that are out there. So I just try to be nice so they won't bother me, so I won't get in trouble or get picked on.” Another participant described missing school due to fear of violence, “Sometimes I won't even go to school, I just walk the other way.” Other focus group members, particularly across the lower‐risk groups, described parental monitoring and travel restriction through certain areas of the neighborhood. Others noted the importance of traveling in groups to ensure safety as well as knowing other members in the community. One student from the lower‐risk college‐bound group described involvement in a program designed to ensure safe passage to and from school.

## DISCUSSION

This study examined youth perceptions of violence exposure, family functioning, and various neighborhood features among Latino youth living in a high violence, low‐income neighborhood using a mixed‐methods CBPR design. Youth mapping and themes surrounding the widespread occurrence of exposure to community violence suggest that this problem is significant. Nonetheless, themes of resilience, in both familial and community contexts, were also revealed by focus groups. Youth discussed the value and protective nature of family and peer support, social capital, and community engagement to buffer the negative effects of violence exposure within their neighborhood.

### Violence exposure in the context of school and route to school

The current study produced novel findings relating to the perspectives of youth and their perceived safety from violence as they traveled to and from school as well as during school hours. As revealed by focus groups, perceptions regarding safety in school were complicated, only partially supporting the study hypothesis. Previous research has revealed that positive relationships with teachers reduced perceived lack of school safety, and a close relationship between neighborhood satisfaction and school safety perceptions has also been found (Peguero et al., 2018). Though several youth described their school and teachers as sources of important socioemotional support, others depicted school grounds as regions of pervasive violence. While the youth generated maps indicated high reports of violence near schools, rates significantly higher than the CPD reports, the maps made by the youth also revealed a safe buffer zone in the blocks directly surrounding schools. However, a sharp increase in reported violence was found in the blocks just outside these safe areas. Furthermore, several participants described racial tension and occasional physical confrontations between African American and Latino students during and immediately following school hours, which is consistent with past literature (Armstead et al., 2021). Using a similar sample, another study found that school, both in and around, is the location in which most daily violence exposure occurred, regardless of whether it was during the week or on the weekend (Richards et al., 2015). Thus, while schools appear to be sources of refuge for which to process neighborhood stressors for some youth, exposure to violence within and around school, for certain neighborhoods, complicates the notion of schools as safe zones for others.

As predicted and in support of previous literature (Kann et al., 2018), it was revealed that exposure to community violence en route to school represents a significant barrier for the current sample. In fact, the mixed‐methods approach of focus group interviews and youth‐mapped school routes revealed that approximately half (53%) of the sample traverse perceived unsafe or gang territories and experience fear for their safety during their commute to school. It was also apparent that feelings of safety altered dynamically, depending on companions, time of day, and neighborhood area. The rate of violence reported in this region as identified by kernel density maps for both CPD and youth‐report reflects that at least some of the fear experienced by the youth is warranted. This is further alarming as 70% of the current sample walk or bike to school, prolonging their potential exposure during their commute. While Safe Passage programs are in place for some of the schools attended by the current study's participants, federal support for the provision of safe routes for students has been losing support in various legislatures (Safe Routes to School National Partnership, 2016). This is in spite of local evidence that violent crime is decreasing along these routes (Chicago Sun Times, 2018). The results of the current study indicate a need for broad and targeted policies to address the safety of youth commuting to and from schools. Additionally, it is critical that youth perceptions of violence are addressed and utilized in the evaluation of Safe Passage programs and policies, as the GIS data of this study indicate youth reports of community violence differ from CPD reports, especially around schools.

### The role of social capital in the context of violence exposure

In addition to schools, other aspects of social capital, including community assets, were found to serve a complicated role within the eyes of youth in the context of exposure to community violence. While youth in the current study described parks as valuable assets within the community and sources of neighborhood satisfaction, which is reflective of previous research (Hart & Mueller, 2013), the same youth noted that these regions emerged as locations of risk. Using a daily sampling approach and a similar socioeconomic, but African American sample, Richards et al. (2015) reported that a disproportionate number of violent incidents were reported in settings of less structure and supervision, such as parks. In contrast to unstructured assets, youth noted the value of after‐school program involvement, volunteering, and church involvement. Notably, no significant spatial relationship was found between any of these community asset variables and either youth‐reported violence or CPD violent crime, reflecting the complex role that these assets may serve in preventing violence exposure. Furthermore, spatial analyses revealed higher reports of violence around organized programs than around parks, including in the relative space buffer zones outside organized programs. Ciorici and Dantzler (2019) also found that families residing within economically disadvantaged and higher‐risk neighborhoods did not report positive evaluations of community activities and the neighborhood as a whole.

Participation in extracurricular activities, however, has been previously linked with a host of positive developmental variables, including academic performance and psychological well‐being (Bohnert et al., 2009; Larson & Brown, 2007). Extracurricular (supervised and structured) involvement, in general, has also been associated with fewer externalizing behaviors and reduced exposure to community violence (Richards et al., 2004). Indeed, many of the youth in the current study indicated that these organizations are means of empowerment and opportunities to demonstrate leadership in their school environment and community. Similar to schools, the youth generated maps revealed a safe buffer zone in the one or two blocks community organizations and a sharp increase in reported violent incidents were observed just outside the buffer zone. Unfortunately, participation can also mean youth are traveling home later in the day and thus, may be exposed to more violence, as most violent crime occurs during after‐school hours (Salzinger et al., 2002).

Another source of social capital, peer support and informal leisure activity, represented a mixed role in experiencing or reducing violence in the lives of these youth. Youth described social support as an integral aspect of navigating their neighborhood, which is in keeping with previous research demonstrating a link between social capital and neighborhood well‐being (Ungar, 2011). The youth described traversing and interacting with the neighborhood as a social unit, in that they relied on strength in numbers to avoid violence and aggressive confrontation. In fact, the GIS mapping data indicated that approximately half (44%) of protective and safe areas in the neighborhood (outside homes) identified by youth were informal peer hangouts, such as friends' homes, malls, and restaurants.

An additional component of social capital, a child's family, emerged as an important protective factor from the focus groups in preventing violence exposure within the neighborhood. The specific cultural value of *familismo* (Cruz‐Santiago & Ramírez García, 2011), may denote a protective factor in the context of a dangerous neighborhood. *Familismo* involves prioritizing family over individual needs, maintaining a strong sense of loyalty and unity with one's family, and increased reliance on family for social support. Consistent with other research (e.g., Outley & Floyd, 2002), the youth from the current study revealed that several of their parents employed a variety of strategies to minimize danger in their environment, including enforcing curfews, restricting areas of allowable travel, and encouraging traveling within groups.

The findings of the current study should be interpreted with methodical and sampling limitations. First, the relatively small sample size, both in terms of number of adolescents surveyed and neighborhoods examined, potentially introduces bias and reducing generalizability. Consistently, the homogenous sample in terms of ethnicity and geographic location limits external validity and generalizability to other populations. While this specificity enhances the relevance of findings to the local context, future research should examine whether similar dynamics hold across ethnically and geographically diverse communities. Additionally, although the cohorts of youth were predetermined by the community partner, which introduced limitations such as the mixed‐gender nature of the focus groups, this also precluded exploration of potential gender differences in the experience of and response to violence exposure (e.g., Zona & Milan, 2011). Given emerging evidence of gender‐specific resilience processes (e.g., Koposov et al., 2021), future work should explore how gender may shape adolescents' appraisals and coping strategies in the face of neighborhood stressors. Finally, although the CBPR design was a foundational strength in this study, the results are biased to only include the experiences of youth actively involved in a community organization. Moreover, the reliance on qualitative inputs and community‐specific frameworks may limit reproducibility across settings or studies not embedded in participatory designs. Nevertheless, these methods offered a rich, contextually grounded understanding of youth perceptions and stress navigation.

### Future research & implications

Future research would benefit from the continued use of CBPR designs to investigate community violence, its sequelae, and protective processes. The present study demonstrated that CBPR, when paired with GIS and mixed‐methods approaches, can yield meaningful insight into youth perceptions of neighborhood safety and resilience. The partnership with the community organization and the implementation of CBPR provided several significant benefits to the current study, foremost being a fruitful dialogue between community stakeholders and the research team, which offered a unified understanding of the study's purpose and method. The research team aimed to ensure that individuals involved were empowered through the research process by fostering opportunity for both the youth and community leaders to identify the strengths and challenges experienced within their neighborhood (Nelson et al., 2014). Both the research team and the community staff worked with the goal of informing successful programming within the organization to ultimately extend this information to other programs within the Little Village community. Future research should conduct CBPR and mixed‐methods designs using GIS approaches, to facilitate integration of qualitative and quantitative data on neighborhood perceptions and youth experiences.

While the findings are highly relevant to the local context, replication in other regions, with youth from more diverse racial/ethnic backgrounds and different urban or rural contexts, will be essential for understanding how generalizable these processes are. Future work should also consider gender‐stratified data collection and analysis, as emerging literature suggests gender‐specific pathways in coping with violence exposure (Zona & Milan, 2011). Although our focus groups were mixed‐gender, future studies could deepen this line of inquiry to examine how boys, girls, and gender‐diverse youth uniquely experience and respond to community violence.

Researchers conducting similar CBPR mixed‐methods studies may benefit from integrating additional explanatory variables such as gang activity, time of day (Richards et al., 2015), family socioeconomic indicators, and school context to refine models of youth‐reported and police‐reported violence exposure. The reproducibility of CBPR findings may be limited due to the localized, collaborative nature of this study; however, the design can be adapted and recontextualized to suit the needs of other community organizations.

The distinctive use of mixed‐methods CBPR has revealed several potentially useful applications for policy and intervention. A similar GIS approach could be produced and utilized by other violence prevention organizations to track violence rates in an organized manner as well as specifically tailor meaningful interventions based on focus group feedback. Similar methodologies could be used to evaluate safe passage programs to identify specific routes where needed. Furthermore, current findings suggest that mental health providers should be cognizant of the multi‐systemic factors that influence Latino youth living in low‐income communities, including fostering positive coping mechanisms in response to exposure to community violence (Reingle et al., 2013). Interventions should encourage and incorporate involvement in structured after‐school programs as well as target family functioning through the promotion of family cohesion and support in these communities. Finally, as protection from violence during school hours and travel to school is a nationwide concern, findings of the current study support the notion that policies should be adopted to improve neighborhood and school safety by investing more strongly and comprehensively in urban communities of color, and thus reducing crime. The data from this study suggest that this is a necessary approach to improve the quality of life for the youth living in these areas.

## CONFLICT OF INTEREST STATEMENT

The authors declare that they have no conflicts of interest to disclose in relation to this manuscript. None of the authors have any financial or nonfinancial interests that could be perceived as influencing the research reported in this manuscript.
